# The mechanism of sevoflurane post-treatment alleviating hypoxic-ischemic encephalopathy by affecting histone methyltransferase G9a in rats

**DOI:** 10.1080/21655979.2021.1995105

**Published:** 2021-12-07

**Authors:** Weifeng Shan, Yini Wu, Xin Han, Qin Chen, Jimin Wu

**Affiliations:** Department of Anesthesiology, Lishui City People’s Hospital, Lishui, China

**Keywords:** Ischemic hypoxic encephalopathy; sevoflurane, post-treatment, G9a, histone H3 lysine 9 dimethylation, brain-derived neurotrophic factor, nerve growth factor

## Abstract

Hypoxic-ischemic encephalopathy (HIE) is recognized as the main cause of neonatal death, and efficient treatment strategies remain limited. This study aims to investigate the mechanism of sevoflurane (SF) post-treatment in alleviating HIE in rats. The HIE rat model and oxygen-glucose deprivation (OGD) cell model were established, and adeno-associated virus (AAV)-histone-lysine N-methyltransferase EHMT2 (G9a) was transfected after SF treatment. The learning and memory ability and the levels of nerve growth factor (NGF)/brain-derived neurotrophic factor (BDNF) were evaluated and determined. The levels of G9a/histone H3 lysine 9 dimethylation (H3K9me2) and the enrichment level of H3K9me2 in the promoter region of BDNF gene were analyzed. After SF post-treatment, the neurons in cerebral cortex, the learning and memory skills and the contents of NGF/BDNF were increased, while the apoptosis and G9a/H3K9me2 levels were reduced. After overexpression of G9a *in vitro/vivo*, the enrichment levels of H3K9me2 in the promoter region of BDNF were increased, the levels of BDNF were decreased, the neurons were damaged and the learning and memory abilities of HIE rats were impaired. The conclusion is that SF post-treatment can promote the expression of BDNF by inhibiting H3K9me2 on the BDNF gene promoter and inhibiting G9a, thus alleviating HIE in rats.

## Introduction

Hypoxic ischemic encephalopathy (HIE) is the most important cause of morbidity and mortality in full-term newborns [1]. It is a devastating disease that mainly causes damage to neurons and white matter and is one of the leading causes of death in infants [2]. In developed countries, the disease occurs in between 1 and 8 births per 1,000 live births [3]. The pathophysiology of HIE includes oxidative stress, mitochondrial energy production failure, glutamine excitatory toxicity, and apoptosis [4]. Neonatal encephalopathy can cause neurodevelopmental and cognitive impairments in survivors of hypoxic-ischemic injury, with or without functional motor impairments [5]. Hypothermia treatment is the only formally endorsed treatment recommended for HIE, but its applicability in protecting against brain injury is limited [6]. Therefore, research on effective methods to reduce HIE is imminent.

Sevoflurane (SF), as an inhalation anesthetic, has an analgesic effect and neuroprotective effect on fetal perinatal asphyxia, which suggests that anesthetics with neuroprotective effects may be effective in the prevention of HIE [7,8]. According to the literature, SF post-treatment can reduce neuronal damage, cerebral infarction and neuronal apoptosis in rats, and improve the long-term cognitive function of rats [9–11]. In conclusion, SF post-treatment can protect brain neurons in HIE rats and alleviate HIE.

Histone methylation is one of the epigenetic mechanisms of HIE [12]. Histone-lysine N-methyltransferase EHMT2 (G9a) and G9a-like (GLP) proteins are lysine methyltransferases that form a heterodimeric complex capable of monomethylating and dimethylating the lysine 9 of histone H3 (H3K9me1 and H3K9me2) the N-terminal tail [13,14]. Epigenetic regulation by G9a leads to increased expression of histone H3 lysine 9 dimethylation (H3K9me2), which in turn leads to hippocampal-dependent memory impairment [14]. Inhibition of histone methyltransferases SUV39H1 and G9a provides neuroprotection in hypoxic metabolic stress models [15]. However, the role of G9a in HIE is largely unknown.

As an important neurotrophic factor, brain-derived neurotrophic factor (BDNF) is involved in the formation and maintenance of synaptic links, and regulates the release of neurotransmitters [16]. It is a major molecular mediator for the function and morphology of neurons and synaptic plasticity [17]. In the study of the pharmacological mechanism of G9a/GLP on early-onset Alzheimer’s disease in rat, inhibition of G9a/GLP complex could increase the levels of synaptophysin, nerve growth factor (NGF) and BDNF in brain tissues, showing a significant neuroprotective effect on rats [13]. We hypothesized that SF post-treatment may be involved in the alleviation of HIE in rats by inhibiting the expression of histone methyltransferase G9a and regulating the H3K9me2 histone modification level in the promoter region of BDNF gene. However, the brain protective mechanism of SF post-treatment is complex, and the improvement of HIE in neonatal rats by affecting the expression of histone methyltransferase G9a and then regulating H3K9me2 histone modification in the promoter region of BDNF gene has not been reported at home and abroad. Therefore, this study aims to explore the ameliorative mechanism of SF post-treatment on brain injury in rats by establishing HIE rat model *in vivo* and OGD cell model *in vitro*.

## Materials and methods

### Ethics statements

All animal experiments in this study were reviewed and approved by the animal ethics committee of Lishui City People’s Hospital. We had done our utmost to minimize the animal number and pain. All procedures were strictly implemented by the code of ethics.

### Establishment of hypoxic ischemic encephalopathy (HIE) rat model

Sixty Sprague–Dawley (SD) rats (30 days old, weighing 115–140 g) were purchased from Beijing Vital River Laboratory Animal Technology Co. Ltd. [Beijing, China, animal license number: SYXK (Beijing) 2016–0011]. Rats were fed in separate cages in standard animal rooms at 22 ± 1°C with of 30%–40% humidity and free drinking water and diet under the condition of 12-h light/dark cycles (light cycle is between 8:00 and 20:00) [10]. SD rats were divided into the following groups: 1. sham group (N = 12): the left common artery of the rat was exposed, and then rats were put back into the cage after waking up for further feeding for 2 hours, and rats were put into a self-made semi-closed box, and normal air was input into the box for 2 hours; 2. HIE group (N = 12): HIE rat model was established according to the above method [13,18]. The left common carotid artery was ligated in rats under an anatomical microscope. After the rats were awake, they were put back into the cage for further feeding for 2 hours. After the surgery, the rats were put into a self-made semi-closed chamber, and a mixture of 8% O_2_ and 92% N_2_ was continuously input into the box at a flow rate of 2 L/min for 2 hours; 3. HIE + SF group (N = 12): After the establishment of HIE model, the rats were immediately inhaled with a mixture of 8% O_2_ and 92% N_2_ containing 2% SF (Shanghai Xiyuan Biotechnology Co. Ltd, Shanghai, China) for 30 minutes; 4. HIE + SF + G9A overexpression adeno-associated virus (AAV) (Bainway Biotechnology Co. Ltd, Shenzhen, China) group (HIE + SF + AAV-G9a, N = 12): 3 uL AAV-G9a solution was injected into the lateral ventricle of the rat 30 minutes before the establishment of the HIE model [19]. The mixture of 8% O_2_ and 92% N_2_ containing 2% SF was inhaled immediately by the rat after the establishment of the HIE model for 30 minutes; 5. HIE+ SF + adeno-associated virus vector (HedgehogBio Science and Technology Ltd., Shanghai, China) group (HIE + SF + AAV-NC, N = 12): 3 uL adeno-associated virus negative control vector was injected into the lateral ventricle of the rat 30 minutes before the establishment of HIE model, and the other procedures were the same as those in the HIE + SF + AAV-G9a group. The rats in the HIE group and sham group were injected with the same amount of normal saline into the lateral ventricle. The rats in each group were firstly subjected to behavioral experiments, followed by intraperitoneal injection of 2% pentobarbital sodium (150 mg/kg). Then the chest of rats was opened. The left ventricle of the heart and the ascending aorta were firstly infused with 20 mL normal saline and then infused with 20 mL 4% paraformaldehyde. The brain tissues of 6 rats in each group were rapidly and randomly selected for hematoxylin-eosin (HE) staining and Nissl staining. The left hippocampus was subjected to TUNEL staining, and the brain tissues of the other 6 rats in each group were used for the quantitative reverse transcription polymerase chain reaction (qRT-PCR), Western blot, enzyme linked immunosorbent assay (ELISA), and chromatin immunoprecipitation technology (CHIP).

### Longa score

Longa score test was performed on rats in the sham group, HIE model group and HIE + SF group at 1 hour before modeling and 7, 14, 21 and 28 days after modeling. Neurobehavioral examination results consisted of 5 grades: 1, the rats had normal behaviors without neurological impairment, and the score was 0; 2, the left forepaw of the rats could not be fully extended, and there was slight neurological impairment, with a score of 1; 3, during walking, the rats turned to the left side (the paralyzed side) and had moderate neurological impairment, with a score of 2; 4, during walking, the body of the rats was tilted to the left side (the paralyzed side), with severe neurological impairment, with a score of 3; 5, the rats cannot walk spontaneously or lose consciousness, and the score was 4 [20].

### Open field test (OFT)

At 28 days after the HIE modeling, OFT were conducted on rats in the sham group, HIE group and HIE + SF group. A square open box of 100 cm × 100 cm × 40 cm was used in the experiment. The bottom of the box was divided into 3 × 3 small squares. The squares along the side wall were the peripheral area, and the rest was the central area. The rats were put into the central square of the open box, and the routes to explore the peripheral area and the central area and the time to stay in the central squares were tracked by the image automatic monitoring system (Shanghai XinRuan Information Technology Co. Ltd., Shanghai, China) within 15 minutes. After each rat finished the test, the field was wiped with 75% alcohol to avoid the residual odor of the previous rat affecting the experimental results [21,22].

### Morris water maze (MWM) test

After the OFT, MWM test was performed on rats in the sham group, HIE model group and HIE + SF group. A circular stainless steel pool with a diameter of 120 cm and a height of 60 cm was divided into four quadrants on average. In the center of the target quadrant, a circular hidden platform with a diameter of 10 cm was placed 1–2 cm beneath the water surface, and the water temperature was controlled at 23–25°C. An image automatic monitoring system (Shanghai XinRuan Information Technology Co., Ltd., Shanghai, China) was installed above the pool. The experiment was divided into training phase and test phase, and the training phase lasted for 5 days and the test phase lasted for 1 day. The day before training, rats in each group were put into a pool (without platform) to move freely in 90 seconds, so that they could get familiar with the pool environment. During the training, the rats were put into the pool from four quadrant walls one by one at 9:00 am every day, and forced into the water to find the target platform. The image self-monitoring system recorded the time when the rats climbed from the water to the target platform, which was the escape latency. The rat stayed in the water for 90 seconds at a time, and after finding the target platform, it stayed on the platform for 20 seconds. If the rat cannot climb on the target platform within 90 seconds, it was guided to climb on the target platform, and the time was recorded as 90 seconds. The rats were trained four times a day at an interval of 60 minutes and the escape latency per day was averaged from 4 trials. After training for each day, the water maze was cleaned and replaced with new water to eliminate the influence of the environment on the experiment. On the 6th day, the target platform in the pool was removed for space exploration test. The rats were put into the water from the opposite quadrant of the original target platform. Then the time for the rat reached the original target platform area was recorded and the times the rat crossing the original target platform area within 90s were also documented [18,23].

### HE staining

The cerebral cortex of rats was washed with precooled phosphate buffer saline (PBS), fixed with 10% neutral formaldehyde at 4°C for 24 hours, and embedded in paraffin. The paraffin-embedded cerebral cortex was cut into 4 μm sections and soaked in warm water at 40°C for 10 minutes, and then dewaxed twice with xylene I (14936–97-1, Shanghai Research Biological Technology Co., Ltd., Shanghai, China) and xylene II (523–67-1, Shanghai Yuduo Biotechnology Co. Ltd., Shanghai, China). The samples were soaked in 100%, 100%, 95%, 95% and 80% ethanol for 5 minutes, respectively, and then washed with PBS. Then, the sections were stained with hematoxylin (C1411-100, Huaxia Ocean Technology Co., Ltd, Beijing, China) for 15 minutes, washed with distilled water for 15 minutes, differentiated with 1% hydrochloric acid ethanol, washed with double steam water for 3 minutes, put into weak ammonia water for 1 minute and washed with water for 10 minutes to make the sections blue. Next, the sections were stained with eosin (CD019, Zhongke Maichen Technology Co. Ltd., Beijing, China) for 10 minutes and rinsed with distilled water. Then, the sections were dehydrated with ethanol gradient, cleared with xylene, and sealed with sealing resin (YT2494, YITA Biotechnology Co., Ltd, Beijing, China). Morphological image analysis system JD801 (JD801, Jeda Technology Co. Ltd., Nanjing, China) was used to observe the morphological changes of neurons after HE staining in cerebral cortex of different groups of rats, and the images were randomly collected [24,25].

### Nissl staining

The cerebral cortex of rats was taken and the residual blood was removed with precooled PBS. The cerebral cortex of rats was fixed in 4% paraformaldehyde at 4°C for 48 hours, then dehydrated by gradient ethanol, embedded in paraffin, and sliced into 3 um sections. After dewaxing, sections were incubated with Nissl staining solution A (ETA biology Co. Ltd., Beijing, China) at 60°C for 1 hour in the dark, and then rinsed with deionized water. Sections were differentiated in Nissl staining solution B (ETA biology) until the background was nearly colorless under the microscope. Finally, the sections were sealed with neutral gum (YITA Biotechnology). Five fields were randomly selected from each section, and the software Image J was used to analyze the number of neurons [26].

### TUNEL staining

The left hippocampus of rats was taken and the residual blood was removed with precooled PBS solution. Tissues were fixed with 4% paraformaldehyde for 24 hours, embedded in paraffin (YITA Biotechnology), and sliced at 3 um. The paraffined sections were dewaxed and treated with sodium citrate buffer (Solarbio, Beijing, China) in a microwave oven for 7 minutes and 40 seconds for antigen repair. The sections were sealed in PBS solution containing 10% fetal bovine serum (Sinobio Chemistry Co. Ltd., Anhui, China) at room temperature for 30 minutes, and then incubated with terminal deoxynucleotide transferase (TdT) and deoxyuridine triphosphate (dUTP) at 37°C for 1 hour in the dark. After 6-diamidino-2-phenylindole dihydrochloride (DAPI) staining for 5 minutes in the dark, sections were washed with PBS solution 3 times, 5 minutes each. Five fields were randomly selected from each section under a fluorescence microscope, and the number of TUNEL-positive cells, namely the number of neuronal apoptosis, was calculated using Image J software [27].

### Cell treatment and grouping

The PC12 cells purchased from Sunncell Biotechnology Inc. (Wuhan, China) were cultured in Dulbecco’s modified Eagle medium (DMEM) supplemented with 10% fetal bovine serum (FBS) and 1% penicillin–streptomycin solution in an incubator with 5% CO2 at 37°C. The medium was replaced every 3 days. Cells at passage 3 were inoculated in glucose-free DMEM without FBS and penicillin–streptomycin solution at a density of 5 × 105/mL in a hypoxic incubator with 1% O_2_ for 3 h, and then cultured in complete growth medium for 24 h to establish the cell model of OGD. OGD cells were transfected with AAV-NC and AAV-G9A, respectively, and cultured in an incubator with 5% CO_2_ at 37°C for 24 h for subsequent experiments.

Cells were grouped as follows: blank group (PC12 cells without any treatment), OGD group, OGD + SF group [OGD-induced PC12 cells were cultured with 2% SF for 30 min [28], OGD + SF + AAV-NC group [OGD-induced PC12 cells transfected with AAV-NC (1 × 10^9^ TU) were cultured with 2% SF for 30 min], OGD + SF + AAV-G9a group [OGD-induced PC12 cells transfected with AAV-G9a (1 × 10^9^ TU) were cultured with 2% SF for 30 min].

### Quantitative reverse transcription polymerase chain reaction (qRT-PCR)

The brain tissues or cell samples of rats were taken and washed with aseptically precooled PBS solution 3 times. The total RNA was extracted according to the instructions of TRIzol (YITA Biotechnology), and then reverse transcribed into cDNA according to the instructions of reverse transcription kit (YITA Biotechnology). Then fluorescence quantitative PCR was performed. The reaction system was 20 uL, including 10 uL of SYBR, 0.8 uL each of PCR forward and reverse primers, 2.0 uL of cDNA template, 6.0 uL of sterilized distilled water, and 0.4 uL of ROX. PCR primers are shown in Table 1. Reaction conditions were as follows: pre-denaturation at 95°C for 5 minutes, and 40 cycles of denaturation at 95°C for 45 seconds, annealing at 60°C for 34 seconds, extension at 72°C for 30 seconds. The CT value of each sample was obtained. The relative expression of the target gene was calculated by the 2^−ΔΔ CT^ method.

**Table 1.** Primer sequences for RT-PCR

Gene	Forward (5ʹ-3ʹ)	Reverse (5ʹ-3ʹ)
*G9a*	GAGGTGTACTGCATAGATGCC	CAGACGGCTCTGCTCCAGGGC
*GAPDH*	GAAGGTGAAGGTCGGAGTC	GAAGATGGTGATGGGATTTC

### Western blot (WB)

The rat brain tissue was washed with pre-cooled PBS to remove the residual blood, weighed and cut into pieces on ice. The precooled tissue lysis buffer was added at the ratio of 10 uL/mg. After the homogenization using an ultrasonic crusher, the tissue sections were centrifuged at 14,000 r/min for 15 minutes at 4°C. The supernatants were placed in centrifuge tubes and stored in a refrigerator at −80°C. After the cell samples were rinsed with pre-cooled PBS, lysed with radioimmunoprecipitation assay reagent (Solarbio) kit, and subpacked in centrifuge tubes, and then stored in a refrigerator at −80°C. The protein in brain tissue lysate or cell sample lysate was quantified by the bicinchoninic acid assay, and the samples were diluted with the same amount of lysis buffer. After separation by sodium dodecyl sulfate-polyacrylamide gel electrophoresis, the samples were transferred to polyvinylidene fluoride membranes at 200 mV constant voltage. The membranes were sealed with 5% skim milk at room temperature for 2 hours, and incubated with the primary antibodies Anti-EHMT2/G9a and Anti-Histone H3 (dimethyl K9) (ab185050, 1:1000, Abcam, Cambridge, UK; ab176882, 1:1000, Abcam) overnight at 4°C. The next day, the membranes were washed 3 times in the shaker, each for 5 minutes, and then incubated with the secondary antibody goat-anti rabbit lgG H&L (ab205718, 1:2000, Abcam) at room temperature for 1 hour. The membranes were washed 3 times in the shaker, each for 5 minutes, and then added with luminescent solution for development on the machine. β-actin (ab8227, 1:1000, Abcam) was used as an internal reference. Image J software was used for gray value analysis to analyze the levels of G9a and H3K9me2.

### Enzyme linked immunosorbent assay (ELISA)

The rat brain tissue was washed with precooled PBS solution to remove the residual blood, weighed and cut into pieces on ice. The precooled tissue lysis buffer was added at the ratio of 10 uL/mg. After the homogenization by ultrasonic crusher, the tissues were centrifuged at 14,000 r/min for 15 minutes at 4°C. The supernatant was put into the centrifuge tubes and placed in a refrigerator at −80°C for storage. According to the instructions of brain-derived neurotrophic factor (BDNF) ELISA kit (China Ocean Technology Co. Ltd., Beijing, China) and nerve growth factor (NGF) ELISA kit (China Ocean Technology), the contents of BDNF and NGF in rat brain tissue were detected.

### Chromatin immunoprecipitation technology (CHIP)

Brain tissue of rats was collected and rinsed with precooled PBS to remove residual blood, cut up on ice. CHIP cell lysis buffer was then added and each tissue sample was manually ground into a single-cell suspension for further use. Cell samples were washed with pre-cooled PBS. CHIP buffer was added into single-cell suspension of brain tissue or cell sample for ultrasonic lysis to obtain ideal chromatin fragments of 150–1000 bp. Immunoprecipitation reaction was then carried out. After treatment overnight with magnetic beads and antibodies (ab1220, 2:25, Abcam), de-cross-linking was carried out. Purified DNA was obtained using a DNA purification kit (DP214-02, Baolinke biotechnology, Beijing, China) and qPCR was performed to assess the enrichment level of H3K9me2 at the binding site of BDNF promoter, and total chromatin was used to standardize the changes. Rabbit immunoglobulin (IgG) antibody (ab172730, Abcam) was used as a negative reference, and primers were designed for the BDNF promoter region: BDNF-F5ʹ-3ʹ: TGATCATCACTCACGACCACG; BDNF-R5ʹ-3ʹ: CAGCCTCTCTGAGCCAGTTACG. The reaction conditions were as follows: pre-denaturation at 95°C for 3 minutes, and 40 cycles of denaturation at 95°C for 10 seconds, annealing at 55°C for 30 seconds, and extension at 62°C for 20 seconds. PCR products were electrophoresed on 1% TAE agarose gel and repeated 3 times [29].

### Statistical analysis

All data were statistically analyzed and plotted using GraphPad Prism 8.0.1 (GraphPad Software Inc., San Diego, CA, USA) and SPSS 21.0 (IBM Corp., Armonk, NY, USA). Data were presented as mean ± standard deviation and in line with normal distribution confirmed by the W test. Unpaired *t* test or one-way repeated measurement analysis of variance (ANOVA) was used to compare the data between the two groups. One-way ANOVA was used for data comparison among groups, and Tukey’s was used for post hoc test. *P* value was obtained from a bilateral test, and a value of *P* < 0.05 indicated the difference was statistically significant

## Results

In this study, we established a rat model of HIE. After the HIE rats were post-treated with SF, the Longa scores of the rats were decreased, the escape latency was shortened, and the times of crossing the original target platform area were increased. Vacuole-like changes of rat neurons was occasionally seen, the nucleus pyknosis was reduced, and the morphology and structure tend to be normal. The number of neurons in cerebral cortex was increased, the number of neurons apoptosis in hippocampal was decreased, and the levels of NGF and BDNF in brain tissue were increased. In addition, SF post-treatment can alleviate neuron injury in HIE rats and down-regulate G9a expression in neurons. SF post-treatment mediated G9a reduced H3K9me2 histone modification level in the promoter region of BDNF gene, increased BDNF level, alleviated brain neuron injury, and improved learning and memory ability of rats.

### Ischemia and hypoxia led to the damage of neuron structure and function in rat brain

**Figure 1.**
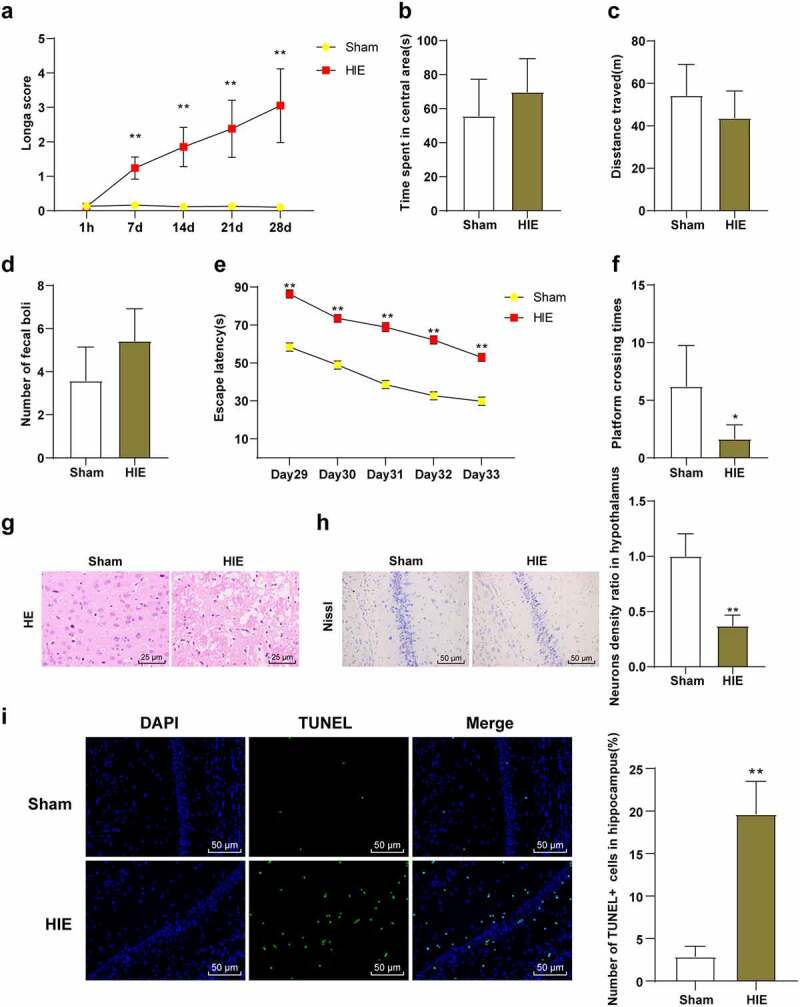
Neurons in brain tissue were damaged in HIE model rats. (a): Longa score of each group of rats, N = 12; (b–d): The central residence time, total distance and feces number of rats in each group in the OFT, N = 12; (e–f): The escape latency and times of crossing the original target platform area in the MWM experiment of rats in each group, N = 12; (g): rat model of HIE was established. (h): HE staining of cerebral cortex of rats in each group, N = 6; (i): Nissl staining of cerebral cortex of rats in each group, N = 6; (d–e): TUNEL staining of the left hippocampus of rats in each group, N = 6. Data were presented as mean ± standard deviation, one-way repeated measurement ANOVA was used in Figure A and Figure E, and unpaired *t* test was used for data comparison between other groups. * *P* < 0.05; ** *P* < 0.01, compared with the sham group

We observed the behavioral changes of rats in the sham group and the HIE group and found that Longa score of rats in the HIE group was higher than that in the sham group (Figure 1a, *p* < 0.01). The OFT showed that there were no significant differences in central stay time, total distance, and fecal number between the sham group and HIE group (Figure 1b–d, *P* > 0.05), indicating that autonomous exploration ability and emotional stress were consistent. MWM showed that the escape latency of HIE group was longer than that of the sham group (Figure 1e, *p* < 0.01), and the times of crossing the original target platform area were reduced (Figure 1f, *p* < 0.05). HE staining showed that the cerebral cortex neurons in the sham group were closely arranged and orderly, with clear and large nuclei and less cytoplasm, and the structure was normal. In the HIE group, the cells were disordered and sparse, the cell body was swollen, the neurons had nuclear pyknosis, the nucleus were irregular, the cell morphology was different, and the loss of cytolysis was obvious (Figure 1g). Nissl staining showed that the number of neurons in cerebral cortex of rats in HIE group was significantly reduced, with partial dissolution or disappearance of Nissl bodies and shallow cytoplasmic staining (Figure 1h, *p* < 0.01). TUNEL staining showed that the number of apoptotic hippocampal neurons in HIE group was significantly higher than that in sham group (Figure 1i, *p* < 0.05). These results indicated that the HIE rat model was established successfully, and the neurons in the HIE rats were damaged, the number of surviving neurons was decreased, the number of apoptotic neuron was increased, and the neurological function and learning and memory ability were impaired.

### SF post-treatment alleviated nerve injury in HIE rats

**Figure 2.**
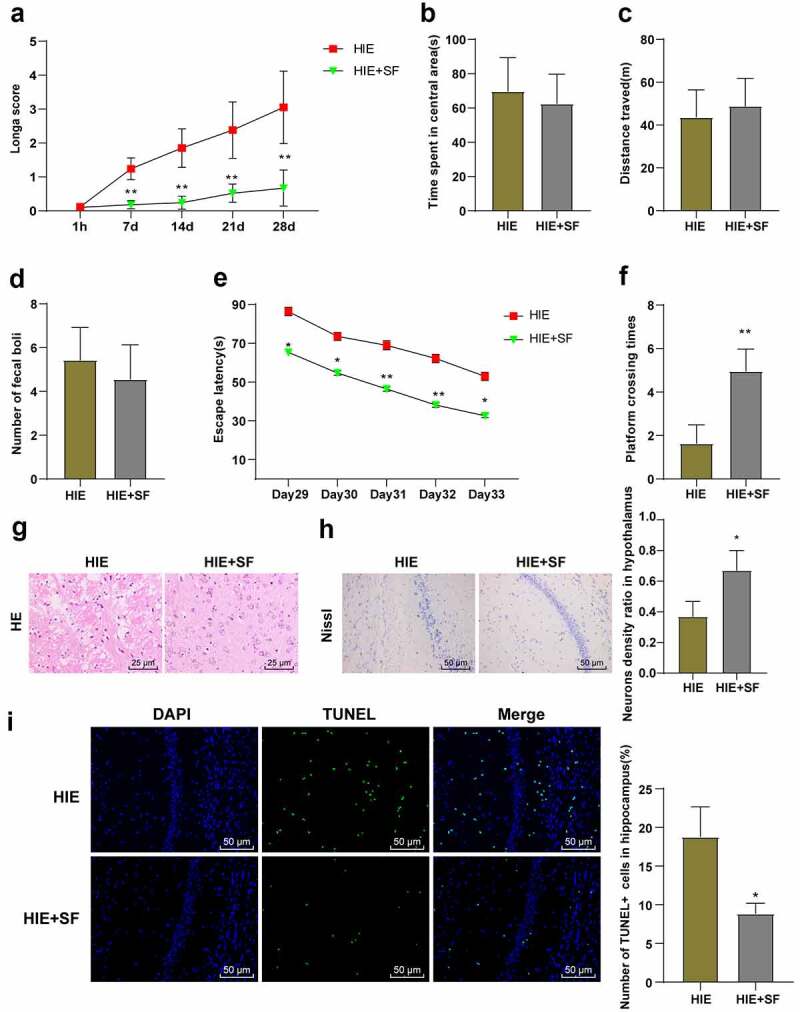
SF post-treatment alleviated neuronal injury in HIE rat model. A rat model of HIE was established and post-treated with SF. (a): Longa score of rats in each group, N = 12; (b–d): The central residence time, total distance and feces number of each group in OFT, N = 12; (e–f): The escaping latency and the times of crossing the original target platform area in the MWM experiment of rats in each group, N = 12. (g): HE staining of cerebral cortex of rats in each group, N = 6; (h): Nissl staining of cerebral cortex of rats in each group, N = 6; (i): TUNEL staining of the left hippocampus of rats in each group, N = 6. Data were presented as mean ± standard deviation, one-way repeated measurement ANOVA was used in Figure A and Figure E, and unpaired *t* test was used for data comparison between other groups. * P < 0.05; ** P < 0.01, compared with the HIE group

We observed the behavioral changes of rats in the HIE group and HIE + SF group and found that Longa score showed a significant decrease in the HIE + SF group (Figure 2a, p < 0.01). The OFT showed that there were no significant differences in central residence time, total distance, and fecal count between the HIE group and HIE + SF group (Figure 2b–d, *P* > 0.05). MWM test showed that the escape latency was reduced in the HIE + SF group (Figure 2e, *p* < 0.01), and the numbers of crossing the original target platform area were increased (Figure 2f, *p* < 0.01). In addition, we examined pathological changes in rat neurons. HE staining showed that in the HIE group, neurons were disordered and sparse, cell body was swollen, neurons had nuclear pyknosis, nucleus were irregular, cell morphology was different, and cytolysis was obviously absent. In the HIE + SF group, vacuolar changes were occasionally observed, nuclear pyknosis was significantly reduced, and both neuron morphology tended to the normal level (Figure 2g). Nissl staining results showed that compared with the HIE group, the number of neurons in cerebral cortex in the HIE + SF group was increased (Figure 2h, *p* < 0.05). TUNEL staining results showed that the number of apoptotic hippocampal neurons was decreased in the HIE + SF group compared with the HIE group (Figure 2i, *p* < 0.05). These results indicate that SF post-treatment can improve the damage of neurons in HIE rats, and can improve the neural function and learning and memory ability.

### Post-treatment with SF decreased the levels of G9a and H3K9me2 in the brain tissues of HIE rats

**Figure 3.**
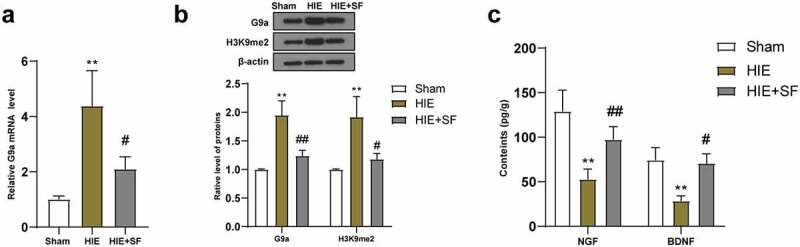
SF post-treatment decreased the levels of G9a and H3K9me2 in the brain tissues of HIE rats. A rat model of HIE was established and post-treated with SF. (a): The mRNA level of G9a in brain tissue of rats in each group was detected by RT-PCR; (b): The levels of G9a and H3K9me2 in brain tissues of rats in each group were determined by WB. (c): The contents of NGF and BDNF in brain tissue of rats in each group were determined by ELISA. N = 6, data were presented as mean ± standard deviation, one-way ANOVA analysis was used for data comparison between groups, and Tukey’s test was used for the post hoc test. * *P* < 0.05; ** *P* < 0.01, compared with sham group; # *P* < 0.05, ## *P* < 0.01, compared with the HIE group

It has been reported that inhibition of the G9a/GLP complex during epigenetic regulation can increase the levels of NGF and BDNF in brain tissues and thus has neuroprotective effects [13]. The expression of G9a can lead to increased expression of H3K9me2, which in turn leads to gene silencing and hippocampal-dependent memory impairment [14]. Subsequently, qRT-PCR was used to detect the mRNA expression of G9a in the brain tissues of rats in the sham group, HIE group and HIE + SF group, and the results showed that G9a expression in the HIE group was higher than that in the sham group; compared with the HIE group, the G9a mRNA expression in the brain tissues of rats in the HIE + SF group were significantly decreased (Figure 3a, both *P* < 0.05). WB results showed that the levels of G9a and H3K9me2 in the HIE group were significantly higher than those in the sham group, and the protein levels of G9a and H3K9me2 in the HIE + SF group were clearly lower than those in the HIE group (Figure 3b, *p* < 0.01 or *P* < 0.05). ELISA results showed that the levels of NGF and BDNF in brain tissues of rats in the HIE group were lower than those in the sham group, and the levels of NGF and BDNF in the HIE + SF group were higher than those in the HIE group (Figure 3c, *p* < 0.01 or *P* < 0.05). In conclusion, SF post-treatment decreased the levels of G9a and H3K9me2 in HIE rats

### SF post-treatment mediates G9a to reduce H3K9me2 in BDNF gene promoter and increase BDNF

**Figure 4.**
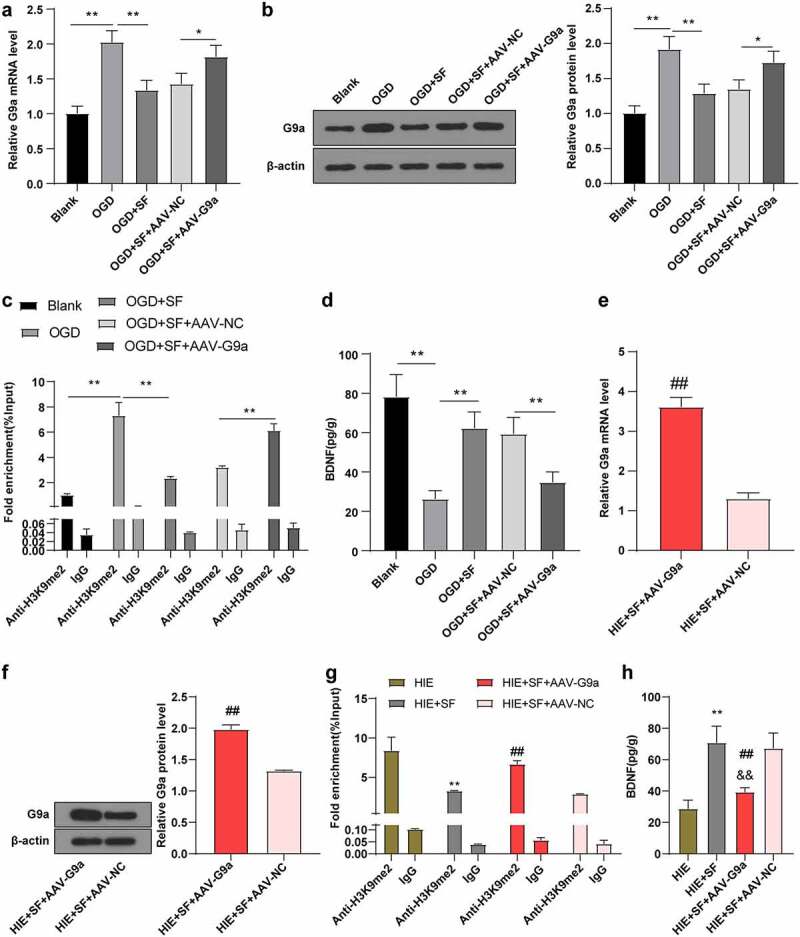
SF post-treatment mediated G9a to reduce BDNF gene promoter H3K9me2 and increase BDNF level. The OGD cell model was established *in vitro* and the OGD cells treated with SF were transfected with AAV-G9A. (a): The mRNA level of G9a was detected by RT-PCR. (b): WB was used to determine the level of G9a in each group. (c): The enrichment level of H3K9me2 in the promoter region of BDNF gene was detected by CHIP. (d): The content of BDNF in each group was determined by ELISA. Then, rats were infected with AAV-G9a vector, and a rat model of HIE was established and post-treated with SF. (e): The mRNA expression of G9a in each group was detected by RT-PCR. (f): The expression of G9a in each group was determined by WB. (g): The enrichment level of H3K9me2 on BDNF gene promoter region was detected by CHIP. He: The content of BDNF in each group was determined by ELISA. N = 6, the cell experiment was repeated three times, data were presented as mean ± standard deviation, one-way ANOVA analysis was used for data comparison between groups, and Tukey’s test was used for the post hoc test. * *P* < 0.05, ** *P* < 0.01

On the basis of the previous experimental results, to further explore the mechanism of SF post-treatment mediated down-regulation of G9a and H3K9me2 and up-regulation of BDNF, we established an OGD cell model *in vitro*. After transfection of AAV-G9A into SF-treated OGD cells, the level of G9a in OGD cells was higher than that in the blank group. The level of G9a in OGD cells was decreased after SF treatment, while after transfection with AAV-G9a, the level of G9a in OGD cells treated with SF was increased (Figure 4a–b, all *P* < 0.05). It has been reported that G9a and G9a-like proteins are the main enzymes expressed by the H3K9me2-downregulated genes [30,31]. Transcriptional levels of downstream target genes are regulated by methylation at different histone sites (e.g., H3K9me2 and H3K27me3) [32]. It has been reported that epigenetic marker H3K9me2 can bind to the promoter region of BDNF gene and reduce the expression of BDNF protein [29]. We speculated that SF post-treatment may reduce the enrichment level of H3K9me2 in the promoter region of BDNF gene by down-regulating G9a level. The CHIP assay was used to detect the enrichment level of H3K9me2 in the BDNF gene promoter, and the results showed that the enrichment level of H3K9me2 in BDNF promoter in cells in the OGD + SF group was lower than that in the OGD group (Figure 4c, *p* < 0.05), the BDNF protein level was increased (Figure 4d, *p* < 0.01); compared with the OGD + SF + AAV-NC group, the enrichment level of H3K9me2 in the BDNF promoter region of cells in the OGD + SF + AAV-G9a group was significantly increased (Figure 4c, *p* < 0.05), and BDNF protein level was decreased (Figure 4d, *p* < 0.05). These results indicate that SF post-treatment can improve the expression of BDNF by down-regulating G9a and reducing the histone modification level of H3K9me2 in the promoter region of BDNF gene. In addition, we found that the same mechanism existed in the brain tissue of HIE rats after SF treatment (Figure 4e–h, all *P* < 0.01).

### SF post-treatment mediates G9a to improve hypoxic ischemic brain injury in rats

**Figure 5.**
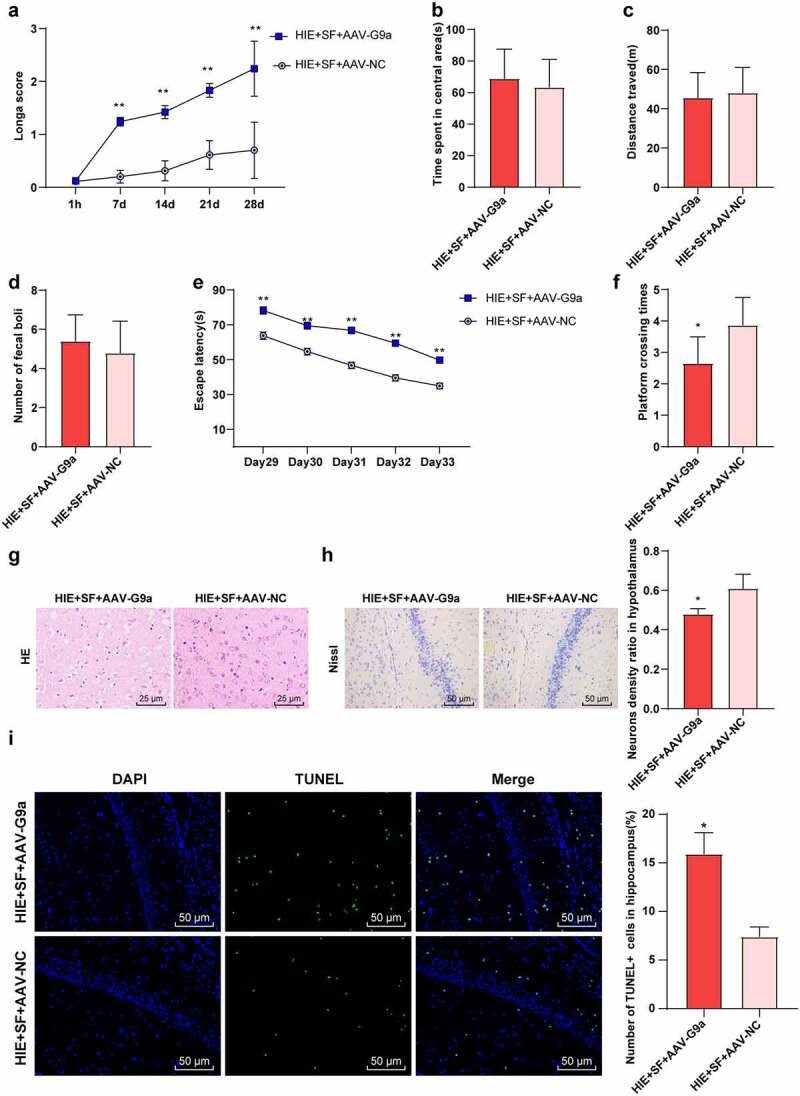
SF post-treatment mediated G9a to reduce HIE in rats. Rats were infected with AAV-G9a vector, and a rat model of HIE was established and post-treated with SF. (a): Longa score of rats in each group, N = 12; (b–d): The central residence time, total distance and feces number of each group in OFT, N = 12; (e–f): The escaping latency and times of crossing the original target platform region in the MWM experiment of rats in each group. (g): HE staining of cerebral cortex of rats in each group, N = 6; (h): Nissl staining of cerebral cortex of rats in each group, N = 6; (i): TUNEL staining of the left hippocampus of rats in each group, N = 6; N = 12. Data were presented as mean ± standard deviation, one-way repeated measurement ANOVA was used in Figure A and Figure E, and unpaired *t* test was used for data comparison between other groups, * *P* < 0.05; ** *P* < 0.01, compared with the HIE + SF + AAV-NC

To further verify whether SF post-treatment can improve brain injury in HIE rats through G9a regulation, we detected the neurobehavioral changes and pathological changes in the HIE + SF + AAV-G9a group and HIE + SF + AAV-NC group. The Longa score showed a significant increase in the HIE + SF + AAV-G9a group (Figure 5f, *p* < 0.01). The OFT manifested that there were no significant differences in central residence time, total distance and feces number between HIE + SF + AAV-G9a group and HIE + SF + AAV-NC group (Figure 5g–i, *P* > 0.05). The MWM experiment demonstrated that the escape latency was increased in the HIE + SF + AAV-G9a group (Figure 5j, *p* < 0.01), and the number of crossing the original target platform was reduced (Figure 5k, *p* < 0.01).HE staining showed that the cells in the HIE + SF + AAV-NC group had less vacuolation, orderly arrangement, and tended to normal morphology, and nuclear pyknosis was significantly reduced. The cells in the HIE + SF + AAV-G9a group had more serious vacuolation, sparse arrangement, different morphology, and neuronal pyknosis with obvious loss of cytolysis (Figure 5a). Nissl staining results showed that compared with the HIE + SF + AAV-NC group, the neurons in cerebral cortex of rats in the HIE + SF + AAV-G9a group were partially dissolved or disappeared, the cytoplasmic staining became lighter, and the number of neurons was decreased (Figure 5b–c, *P* < 0.05). TUNEL staining results showed that the number of apoptotic hippocampal neurons in HIE + SF + AAV-G9a group was higher than that in HIE + SF + AAV-NC group (Figure 5d–e, *P* < 0.05). In conclusion, SF post-treatment may improve HIE in rats by inhibiting G9a expression.

## Discussion

HIE can lead to neurodevelopmental and cognitive impairment in survivors, with or without motor impairment [5] and is considered to be the main cause of neonatal death [24,25]. SF pre-treatment and post-treatment can protect the brain [9], but the neuroprotective effect of SF pretreatment on HIE is limited [26]. In this study, we demonstrate that SF post-treatment can relieve HIE in rats by down-regulating the expression of G9a, inhibiting the H3K9me2 histone modification level on the BDNF gene promoter region, and promoting the expression of BDNF gene.

SF has analgesic and neuroprotective effects on perinatal intrauterine asphyxia, which may be effective in the prevention of HIE [8]. SF post-treatment can relieve the damaged hippocampal neurons after HIE, repair the damaged hippocampal-dependent memory function and spatial learning ability, and has long-term neuroprotective effects [33]. To observe the role of SF post-treatment in HIE rats, the HIE rats were post-treated with SF. Meanwhile, we performed a series of experiments to observe the morphology of cerebral cortex neurons and detect the apoptosis of hippocampal neurons in HIE rats, and assessed the ability of autonomous exploration, emotional tension, spatial learning and memory. We discovered that the nuclear pyknosis was significantly reduced, the number of neurons in cerebral cortex was increased, apoptosis was decreased, the neurobehavioral score of rats was decreased, escape latency was shortened, and the times of crossing the original target platform was increased. These results are consistent with a previous conclusion that SF can improve neurological deficits and reduce apoptotic neurons and thus is neuroprotective in brain damage [34]. Briefly, post-treatment with SF can improve the damage of neurons in HIE rats, and improve the neurological function and learning and memory ability.

Wang et al. [28] demonstrate that SF post-treatment reduced G9a and H3K9me2 protein levels, increased NRPF2 protein levels, alleviated inflammation and neuronal apoptosis, and thus alleviated hypoxic ischemic brain injury (HIBI) in neonatal rats through HIBI models *in vivo* and *in vitro*. The histone methyltransferase regulates the transcription level of downstream target genes through methylation modification at different histone sites (such as H3K9me2 and H3K27me3) [32]. G9a and G9A-like proteins are the main enzymes expressed by H3K9me2 down-regulated genes [30,31]. G9a-mediated demethylation of histone H3K9me2 and H3K27me3 modulates neurodegeneration in the developing brain [35]. Our findings demonstrated that SF post-treatment-mediated G9a reduced the H3K9me2 histone modification level in the promoter region of BDNF gene, elevated the expression of BDNF, and improved neuronal injury, thus alleviating brain injury in HIE rats. G9a and H3K9me2 are increased in the neurons after nerve injury [36]. Inhibition of the G9a/GLP complex can increase the levels of NGF and BDNF in brain tissues and thus produce neuroprotective effects [13]. Epigenetic regulation of the G9a/GLP histone lysine methyltransferase complex is becoming as a key mechanism in learning and memory processes [14,37,38]. H3K9me2 (a marker associated with gene suppression) and K3K9-specific histone methyltransferase G9a were associated with memory consolidation [14,39,40]. After HIE rats were post-treated with SF, the levels of G9a and H3K9me2 were decreased, and the contents of NGF and BDNF were increased. Hippocampal BDNF and NGF levels were obviously decreased in AD rat models and AD patients [41]. Epigenetic modification and neurodegeneration are associated with decreased cognitive ability, due to abnormal transcriptional activity, resulting in neuronal dysfunction [42,43]. These epigenetic changes may be due to the direct involvement of genes encoding proteins in methylation and histone modification, resulting in gene mutation [44]. It is reported that the epigenetic marker H3K9me2 can bind to the promoter region of BDNF gene to reduce BDNF protein level [29]. We speculated that the decreased enrichment of H3K9me2 in the promoter region of BDNF gene might be due to the down-regulation of G9a level by SF post-treatment. Thus, the enrichment of H3K9me2 in the BDNF gene promoter was determined using CHIP assay, which showed that the enrichment of H3K9me2 in the BDNF promoter region was reduced and BDNF protein level was increased. But G9a overexpression reversed the trends. Elevated levels of G9a and H3K9me2 are responsible for hippocampal memory impairment [14]. Consistently, SF post-treatment decreased G9a and H3K9me2 levels in rats with neonatal hypoxic-ischemic brain injury [28]. These results suggested that SF post-treatment can reduce the histone modification level of H3K9me2 in the promoter region of BDNF gene by downregulating G9a, thus increasing BDNF.

In conclusion, this study revealed that SF post-treatment inhibited G9a expression, decreased the H3K9me2 histone modification level in the promoter region of BDNF gene, and increased the expression of BDNF, thus protecting brain neurons and alleviating HIE in rats. However, the main genes and specific molecular mechanisms of the neuroprotective effect of SF post-treatment on HIE are still not completely clear, and a lot of related studies are still needed.

## Conclusion

SF post-treatment reduced the neuron injury in brain tissue of HIE rats, down-regulated the expression of G9a in neurons, reduced the H3K9me2 histone modification level in the promoter region of BDNF gene, increased the level of BDNF, and improved the learning and memory ability of rats.

## Data Availability

All the data generated or analyzed during this study are included in this published article.
